# Treatment of relapsing multiple sclerosis – recommendations of the Croatian Neurological Society

**DOI:** 10.3325/cmj.2022.63.379

**Published:** 2022-08

**Authors:** Mario Habek, Ivan Adamec, Barbara Barun, Vanja Bašić Kes, Andrijana Bogoje Raspopović, Klaudia Duka Glavor, Tereza Gabelić, Tihana Grzinčić, Mirjam Jukić, Miljenka-Jelena Jurašić, Spomenka Kiđemet-Piskač, Milica Komšo, Julija Rimac, Ines Lazibat, Branka Lukić, Anita Marčinko, Meri Matijaca, Marija Ratković, Lidija Šapina, Vladimira Vuletić, Tea Mirošević Zubonja, Magdalena Krbot Skorić

**Affiliations:** 1 Department of Neurology, University of Zagreb School of Medicine, Zagreb, Croatia; 2 University Hospital Center Zagreb, Zagreb, Croatia; 3 Department of Neurology, Sestre Milosrdnice University Hospital Center, Zagreb, Croatia; 4 Department of Neurology, General Hospital Dubrovnik, Dubrovnik, Croatia; 5 Department of Neurology, General Hospital Zadar, Zadar, Croatia; 6 Department of Neurology, General Hospital Bjelovar, Bjelovar, Croatia; 7 MS Team Croatia, Croatia; 8 General Hospital Varaždin, Varaždin, Croatia; 9 Department of Neurology, General Hospital Vukovar, Vukovar, Croatia; 10 Department of Neurology, University Hospital Dubrava, Zagreb, Croatia; 11 Association of Multiple Sclerosis Societies of Croatia, Croatia; 12 Department of Physical Medicine and Rehabilitation, Sveti Duh University Hospital, Zagreb, Croatia; 13 Department of Neurology, University Hospital of Split, Split, Croatia; 14 Department of Neurology, General Hospital Dr. Josip Benčević, Slavonski Brod, Croatia; 15 Department of Neurology, Clinical Hospital Centre Rijeka, Rijeka, Croatia; 16 Department of Neurology, University Hospital Center Osijek, Osijek, Croatia

## Abstract

Untreated multiple sclerosis (MS) irretrievably leads to severe neurological impairment. In European health care systems, patient access to disease modifying therapies (DMT) is often confined to more advanced stages of the disease because of restrictions in reimbursement. A discrepancy in access to DMTs is evident between West and East European countries. In order to improve access to DMTs for people with MS (pwMS) living in Croatia, the Croatian Neurological Society issued new recommendations for the treatment of relapsing MS. The aim of this article is to present these recommendations. The recommendations for platform therapies are to start DMT as soon as the diagnosis is made. If poor prognostic criteria are present (≥9 T2 or FLAIR lesions on the initial brain and spinal cord magnetic resonance imaging [MRI] or ≥3 T1 lesions with postcontrast enhancement on the initial brain and spinal cord MRI or Expanded Disability Status Scale after treatment of the initial relapse ≥3), high-efficacy DMT should be initiated. If pwMS experience ≥1 relapse or ≥3 new T2 lesions while on platform therapies, they should be switched to high-efficacy DMT. Further efforts should be made to enable early and unrestricted access to high-efficacy DMT with a freedom of choice of an appropriate therapy for expert physicians and pwMS. The improvement of access to DMT achieved by the implementation of national treatment guidelines in Croatia can serve as an example to national neurological societies from other Eastern European countries to persuade payers to enable early and unrestricted treatment of pwMS.

Multiple sclerosis (MS) is a chronic, inflammatory, demyelinating, and neurodegenerative disease of the central nervous system (CNS) with a variable and unpredictable clinical course (1). Worldwide there are more than 2 800 000 people with MS (pwMS), while the corresponding number in Croatia is 6500 people (2,3).

Untreated MS irretrievably leads to a severe neurological impairment, including physical and cognitive decline influencing the quality of personal and professional lives. Natural history studies demonstrated a reduced life expectancy in people with MS (pwMS) (4,5).

MS is classified into three major clinical subtypes: relapsing-remitting MS (RRMS) affects 85% of patients. In a proportion of patients, it develops over time to a secondary progressive MS (SPMS), marked with increasing worsening of the disease. Approximately 10–15% of pwMS are affected with a primary progressive MS (PPMS), which is characterized by a continuous deterioration leading to severe ambulation impairment (6).

Disease-modifying therapies (DMT) noticeably affect the inflammatory phase of the disease but they have only a modest effect when introduced in the neurodegenerative and progressive phases (7).

Despite this overwhelming evidence, patient access to DMT in European health care systems is often confined to more advanced stages of the disease because of restrictions in reimbursement regardless of broader regulatory labels (8). A discrepancy in access is evident between West and East European countries. Until 2018, the access to DMT in Croatia was extremely limited, with first-line treatment available only for pwMS who had disease duration longer than one year and at least two relapses treated with steroids in a period of two years. In 2018, the Croatian Neurological Society issued Croatian recommendations for the treatment of MS, which significantly improved the access to DMT (9). This also significantly increased the number of treated pwMS (Figures 1).

**Figure 1:**
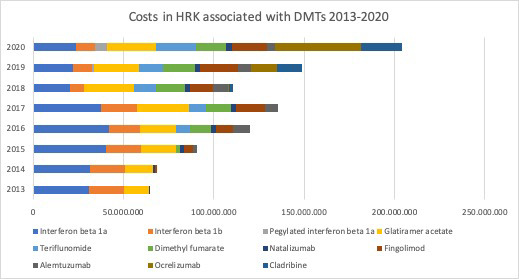
Costs in Croatian kunas (HRK) associated with disease modifying therapies (DMT) for multiple sclerosis (MS), 2013-2020 (Available from: www.halmed.hr, accessed on January 13, 2022). Until 2016, treatment with high-efficacy DMT was not reimbursed by national health insurance. In 2018, the first guidelines of the Croatian Neurological society were published, enabling the initiation of platform therapies as soon as the diagnosis of MS is established. In the same year, the prices of platform DMT were significantly reduced. Note a significant increase in the costs associated with DMT indicating an increase in the number of treated patients with both platform and high-efficacy therapies.

The aim of this article is to present new recommendations of the Croatian Neurological Society for the treatment of relapsing MS. The guidelines are a result of series of in-person meetings, Algorithm in the Treatment of Multiple Sclerosis, held yearly from 2016-2021. Two national MS patients’ associations, MS Team Croatia and Association of Multiple Sclerosis Societies of Croatia, were actively involved in the preparation of the guidelines.

## Recommendations for initiation of treatment of pwMS with platform therapies

The usual treatment strategy in MS includes an escalation approach in which first-line or platform therapies (glatiramer acetate [GA], interferons beta-1a and beta-1b [IFNβ], teriflunomide [TFN], and dimethyl fumarate [DMF]) are introduced at the treatment initiation. These drugs moderately affect MS activity, but are considered safe, given the fact that this kind of treatment requires long term-administration (10).

Randomized clinical trials using GA, IFNβ, and TFM conducted on people with clinically isolated syndrome (CIS) demonstrated a significant delay in conversion to clinically definitive MS according to 2010 McDonald criteria, underlying the importance of early treatment initiation (11,12). GA, IFNβ, and TFM also attenuated brain atrophy in people with CIS (13). The introduction of the 2017 McDonald criteria increased the sensitivity in establishing the diagnosis of MS, enabling early diagnosis and treatment in patients who would be considered as CIS according to 2010 criteria (14).

Randomized controlled trials of GA, INFβ, TFM, and DMF for treatment of people with relapsing MS demonstrated a reduction of relapse rate, MRI disease activity, and, some trials demonstrated a reduction of disability progression compared with placebo (Table 1) (12).

**Table 1:** The main findings from randomized control trials of disease-modifying therapies in multiple sclerosis*

Drug (study)	Comparator	Number of pwMS (study drug/comparator)	Relapse rate reduction (%)	Reduction of the progression (%)	Reduction of the MRI activity (%)	Side-effects	The need for regular monitoring
**Platform therapies – maintenance therapies**							
Interferon beta 1 a (MSCRG study) (43)	Placebo	301	32	37	27 (NS)	Injection site reactions, flu-like symptoms, elevated liver enzymes, depression	Not necessary
Interferon beta 1a (PRIMSM study) (44)	Placebo	560	33	31	78	Injection site reactions, flu-like symptoms, elevated liver enzymes, depression	Not necessary
Interferon beta 1b (MSSG study) (45)	Placebo	372	34	29	83	Injection site reactions, flu-like symptoms, elevated liver enzymes, depression	Not necessary
Glatiramer acetate (CMSSG study) (46)	Placebo	251	29	12 (NS)	35	Injection site reactions, flu-like symptoms, systematic reaction to drug administration	Not necessary
Teriflunomide (TEMSO TOWER study) (47,48)	Placebo	1086/1165	37/32	30/33	69/NA	Gastrointestinal symptoms, hair thinning or decreased hair density, rash, elevated liver enzymes, peripheral neuropathy	Blood pressure, CBC, liver enzymes before the treatment, during the first six months liver enzymes every two weeks, and after that every six weeks
Dimethyl fumarate (DEFINE/CONFIRM study) (49,50)	Placebo/glatiramer acetate	1237/1430	53/44	38/21 (NS)	85/71	Flushing, gastrointestinal symptoms, progressive multifocal leukoencephalopathy (rarely)	Differential blood count before the treatment, and after every two-three months (PML risk – lymphocytes <0.5× 10^9^/L)
**High-efficacy therapies – maintenance therapies**							
Natalizumab (AFFIRM study) (51)	Placebo	942	68	54	83	Infusion reactions, infections, progressive multifocal leukoencephalopathy	JCV titer every 6 months, MR every 3-6 months for patients with JCV positive titer after the second year of treatment
Fingolimod (FREEDOMS 1/2 study) (52,53)	Placebo	1272/1083	54/50	37/28 (NS)	75/74	Bradycardia, AV block, macular edema, increased infection risk (especially VZV) progressive multifocal leukoencephalopathy	Before the treatment: ECG, optic fundus or OCT, CBC, VZV (if the patient was not previously infected); after the treatment: CBC regularly (lymphocytes should be >0.2 × 10^9^/l), optic fundus/OCT after three months
Ozanimod (RADIANCE and SUNBEAM) (54,55)	Interferon beta 1a s.c.	1320/1346	38/48	5 (Pooled analysis, NS)	42/48	Bradycardia, macular edema, increased infection risk (especially VZV) progressive multifocal leukoencephalopathy	Before the treatment: ECG, optic fundus or OCT, CBC, VZV (if the patient was not previously infected); after the treatment: CBC regularly (lymphocytes should be >0.2 × 10^9^/l), optic fundus/OCT after three months
Ponesimod (OPTIMUM) (56)	Teriflunomide	567	31	17 (NS)	56	Bradycardia, AV block, macular edema, increased infection risk (especially VZV) progressive multifocal leukoencephalopathy	Before the treatment: ECG, optic fundus or OCT, CBC, VZV (if the patient was not previously infected); after the treatment: CBC regularly (lymphocytes should be >0.2 × 10^9^/l), optic fundus/OCT after three months
Ocrelizumab (OPERA I and II studies) (57)	Interferon beta 1a s.c.	821/835	46/47	40 (pooled results)	77/83	Infusion reactions, increased infection risk, increased risk of tumors?	CBC, IgG and IgM every six months
Ofatumumab (ASCLEPIOS I and II) (58)	Teriflunomide	927/955	51/59	33 (pooled results)	82/85	Injection reactions, increased infection risk, increased risk of tumors?	CBC, IgG and IgM before every treatment dose
**High-efficacy therapies – Immunoreconstitutional therapies**							
Alemtuzumab (CARE – MS I/CARE – MS II studies) (59,60)	Interferon beta 1a s.c.	578/628	55/48	30 (NS)/41	16/62	Infusion reactions, autoimmune thyroid disease, idiopathic thrombocytopenic purpura, glomerulonephritis, infections	During the treatment and four years after the last treatment dose: CBC, creatinine and urine once a month, TSH every three months
Cladribine (CLARITY study) (61)	Placebo	1326	57.6	33	73.4	Increased infection risk, increased risk of tumors?	Lymphocytes before the treatment, and 2 and 6 months after the beginning of treatment every year

*Abbreviations: pwMS – people with multiple sclerosis; MRI – magnetic resonance imaging; CBC – complete blood count; PML – progressive multifocal leukoencephalopathy; JCV – John Cunningham virus, IgG – immunoglobulin G; IgM – immunoglobulin M; OCT – optic coherence tomography; TSH – thyroid stimulating hormone; VZV – varicella zoster virus, s.c. – subcutaneous; NS – not significant.

Furthermore, real-world evidence studies demonstrated that pwMS treated with IFNβ or GA had a decreased risk of conversion to SPMS compared with untreated patients. pwMS who initiated GA or IFNβ within five years of disease onset had a lower probability of conversion to SPMS compared with those who started the treatment later (15). Another, prospective single-center study, evaluated long-term disability in actively treated pwMS, almost 60% of whom were treated with a platform therapy including IFNβ and GA. At a median of 16.8 years after disease onset, 10.7% of participants reached an EDSS 6.0, while 18.1% converted from relapsing MS to SPMS (16). The rates of conversion to SPMS were significantly lower when compared with previously published natural history studies.

The introduction of DMT significantly changed pregnancy planning in women with MS. One of the strongest risk factors for postpartum relapse is untreated MS two years before conception. IFNβ and GA are allowed during pregnancy, while IFNβ beta is allowed during breastfeeding as well (17).

Taking into consideration published studies, as well as European Academy of Neurology (EAN) and European Committee for Treatment of Multiple Sclerosis (ECTRIMS) guidelines (18), our recommendations for starting platform therapies in people with relapsing MS are as follows:

### Recommendation for initiation of platform therapies (interferon beta/glatiramer acetate/teriflunomide/dimethyl fumarate) in people with relapsing MS:

1. Fulfilled 2017 revised McDonald diagnostic criteria for relapsing-remitting MS

2. EDSS≤6.5

3. Absence of pregnancy (treatment with interferons and GA can be continued during pregnancy if indicated).

## Recommendations for initiation of treatment with high-efficacy therapies

Traditional treatment strategy in MS has been the escalation approach, in which treatment is started with a modestly effective DMT and then escalated to a higher-efficacy drug if there is evidence of clinical and/or radiologic disease activity. Development of higher-efficacy therapies led to another treatment strategy, also known as induction approach, in which high-efficacy medications are started early in the disease course. These medications include infusion therapies (natalizumab, alemtuzumab, and ocrelizumab), cladribine, and ofatumumab. Some experts consider also sphingosine-1-phosphate receptor (S1PR) modulators to be intermediate or higher-efficacy medications (19). High-efficacy medications are given either as maintenance therapy (natalizumab, ocrelizumab, ofatumumab, and S1PR modulators), which is characterized by continuous treatment with a particular medication, or as immune reconstitution therapy comprising of short treatment courses of DMT, which is hypothesized to protect against a breakthrough of disease activity for years after a short treatment course.

Patients with an aggressive or highly active course of MS, who might especially benefit from an early treatment with high-efficacy therapy, are identified based on demographical, clinical, and paraclinical factors.

Of demographical factors, male sex, late disease onset (>40 years), and African American, African, and Latin American origin are risk factors for a poorer prognosis (20). When it comes to clinical risk factors, the most important ones are frequent relapses in the first two to five years with short inter-attack interval and moderate or severe neurological damage (>1 point change on EDSS or >2 point change on any individual functional system score, or >1 point change on any two functional system scores) that require steroids or hospital admission and that are multifocal affecting motor, cerebellar, sphincteric, or cognitive functions with partial or incomplete recovery. Another important clinical risk factor is rapid accumulation of disability (reaching Expanded Disability Status Scale [EDSS] of 3.0 during the first five years with superimposed relapses) (21-23). Finally, MRI signs that present risk factors for a future highly active disease are high T2 lesion burden, the presence of more than two gadolinium-enhancing lesions, T1-hypointense lesions (the so-called black holes), early signs of atrophy, infratentorial lesions at onset, new T2 lesions, and more than one gadolinium-enhancing lesion at follow-up MRI (23). When grading the importance of all these individual risk factors, demographic characteristics represent low-impact prognostic factors, the presence of oligoclonal bands in cerebrospinal fluid is a medium-impact prognostic factor, while more than 10 T2 lesions on initial brain MRI is a high-impact prognostic factor for future disease progression defined as reaching EDSS≥3 (22).

Although no randomized controlled trials have addressed the issue whether an early-initiation of high-efficacy therapies reduces the accumulation of long-term disability compared with a later initiation, there have been several longitudinal observational studies on the issue. A systematic review, which included 12 clinical trials (9 randomized clinical trials and 3 observational studies) using alemtuzumab, natalizumab, and fingolimod with a treatment delay of 0.5–2 years, found that high-efficacy therapies were more efficacious in suppressing disease activity when initiated early vs later in the course of the disease. On the other hand, the evidence regarding disability progression and MRI activity were inconclusive (24). In a study involving 1555 people with RRMS, initial treatment with fingolimod, alemtuzumab, or natalizumab was associated with a lower risk of progression to SPMS than initial treatment with injectables (15). Another study compared five-year disability outcomes between patients with poor prognostic factors (including higher relapse rates and radiological evidence of recent activity) who received an early intensive approach (EIT) (alemtuzumab or natalizumab) (104 of 592 or 17.6%) and patients who underwent an escalation approach (ESC) (488 of 592 or 82.4%). The EIT group had a lower mean five-year change in the EDSS score (25). A study based on the Italian MS register included patients with different types of DMT in the EIT group (fingolimod, natalizumab, mitoxantrone, alemtuzumab, ocrelizumab, cladribine). The ESC group consisted of pwMS who received high-efficacy DMT after more than one year of treatment with GA, IFNβs, azathioprine, teriflunomide, or dimethyl fumarate. Mean annual changes in EDSS values were significantly higher in the ESC group (26). Finally, a study that compared EDSS increase at 6-10 years after disease onset between pwMS who started early (0-2 years after disease onset) high-efficacy therapy (rituximab, ocrelizumab, mitoxantrone, alemtuzumab, or natalizumab) and those who started the same treatment late (4-6 years) found that the early group had a lower mean EDSS score in the sixth year (2.2 vs 2.9, *P* <  · 0001). The difference in the EDSS values between the two groups persisted each year of follow-up until ten years after disease onset (27).

Before starting high-efficacy treatment, it is important to consider the risk of developing side effects. The most common side effects of anti-CD20 cell therapies such as ocrelizumab and ofatumumab include infusion or injection-site reactions. Potential severe adverse events include reactivation of viruses, such as hepatitis B and herpes zoster, tuberculosis, occurrence of progressive multifocal leukoencephalopathy (PML), and development of malignancies (28). A major concern regarding natalizumab treatment is the risk of PML. Depending on the patient’s serostatus, serum John Cunningham virus antibody titers should be monitored every 3–6 months (29). The most common side effect of alemtuzumab administration are infusion-related reactions. Secondary autoimmunity in the form of thyroid disease, idiopathic thrombocytopenic purpura, or antiglomerular basement membrane disease may occur, as well as Listeria monocytogenes and herpes zoster infections (28). Finally, common side effects of cladribine include fatigue and headache, as well as lymphopenia, infections, and increased risk of malignancy (30).

Women of childbearing potential should use reliable contraception while receiving high-efficacy treatment. Women receiving ocrelizumab should use contraception for 12 months after the last infusion (31), while those receiving ofatumumab should use it for six months after the last injection (32). Although natalizumab treatment is generally not recommended during pregnancy in women with highly active MS, the benefit of continuing natalizumab during the entire pregnancy should be weighed against the potential risk of disease activation (33). Cladribine is contraindicated for use in pregnancy and a washout period of at least 6 months following the last dose is required (34). As for alemtuzumab, the recommended washout is 4 months after the last infusion (35).

Taking into consideration published studies, as well as EAN/ECTRIMS guidelines (18), our recommendations for starting high-efficacy therapies in people with relapsing MS are as follows:

### Recommendation for initiation of high-efficacy therapies (natalizumab/S1PR modulators/alemtuzumab/ocrelizumab/cladribine/ofatumumab) in people with relapsing multiple sclerosis:

1. Fulfilled 2017 revised McDonald diagnostic criteria for relapsing-remitting multiple sclerosis

2. One of the following criteria:

a. ≥9 T2 or FLAIR lesions on the initial brain and spinal cord MRI

b. ≥3 T1 lesions with postcontrast enhancement on the initial brain and spinal cord MRI

c. EDSS after treatment of the initial relapse ≥3.

## Recommendations for switching from platform to high-efficacy therapies

PwMS treated with DMT should be carefully monitored for signs of disease activity if we want to properly assess drug efficacy and detect non-responders, who need to be switched to a more potent treatment. Several studies have aimed to establish the level of radiological or clinical activity that may predict the risk of future disease activity. These studies are largely limited to pwMS treated with IFNβs and GA.

The Rio score was designed to identify suboptimal responders to IFN therapy based on clinical and radiological parameters (36). The researchers classified pwMS based on MRI activity (>2 new or enlarging T2 lesions), relapses and confirmed disability progression (an increase in the EDSS of 1) during the first year of treatment and followed them for additional two years. This study demonstrated that the combination of MRI activity along with the presence of relapses (odds ratio [OR] 4.4, 95% confidence Interval [CI] 1.6–12.5) or disability progression (OR 7.1, 95% CI 1.6–33.9), or both (OR 6.5, 95% CI 1.9–23.4), predicted disease activity after three years. A simplified version of the Rio score based solely on relapses and MRI activity, the modified Rio score, was applied in a study by Sormani et al (37). This study followed up pwMS treated with IFNβ for four years. Patients who did not experience a relapse and had ≤4 new T2 lesions in the first year of treatment had a three-year progression probability of 24%. On the other hand, patients who either experienced a relapse or had >4 new T2 lesions had a three-year progression probability of 33% (hazard ratio [HR] 1.56, 95% CI 0.87–2.78, *P* = 0.13), while this probability increased to 65% (HR 4.60, 95% CI 2.51–8.43, *P* < 0.001) for pwMS who experienced relapses and had >4 new T2 lesions. Similar results have been obtained in pwMS treated with GA (38). Patients who experienced relapses and MRI activity during the first year of treatment had an increased risk of continuing with relapses and/or disability progression in the following two years (OR 38.8, *P* < 0.0001).

The Magnetic Resonance Imaging in MS (MAGNIMS) study group performed a multicenter study assessing the association of MRI or relapses with the risk of treatment failure, developing the MAGNIMS score (39). The study included pwMS treated with IFNβ for one year with a further clinical follow-up of at least two more years. The risk of treatment failure was increased in patients who had substantial MRI activity, defined as ≥3 new T2 lesions (HR 1.55, 95% CI 0.92–2.60, *P* = 0.09) and 1 relapse while on therapy (HR 1.84, 95% CI 1.39–2.44, *P* < 0.001). The risk of treatment failure was lowest (17%) in patients without relapses and <3 new T2 lesions (MAGNIMS score 0). In patients with 1 relapse or ≥3 new T2 lesions (MAGNIMS score 1), the risk was 27%, and in patients with both conditions or more than 1 relapse (MAGNIMS score 2), the risk was 48% (*P* < 0.001). MAGNIMS score was also applied to patients who originally participated in a double-blind, randomized, placebo-controlled clinical trial of IFNβ 1a administered subcutaneously in RRMS (40). This enabled the validation of the score in a cohort with a longer follow-up of up to 15 years. The risk of confirmed EDSS progression was higher with a year-1 MAGNIMS score of 1 vs 0 (HR 1.93, 95% CI 1.23–3.02, *P* < 0.0001); 2 vs 0 (HR 2.95, 95%CI 1.95–4.46, *P* < 0.0001); and 2 vs 1 (HR 1.53, 95% CI 1.05–2.23, *P* < 0.0001). MAGNIMS score was also validated for other DMTs besides IFNβ. A post-hoc analysis was performed in a subgroup of pwMS who received teriflunomide in the original double-blind, randomized, placebo-controlled clinical trial of teriflunomide in RRMS (41). Patients with a MAGNIMS score of 2 after one year of the treatment had a significantly higher risk of confirmed disability progression over seven years than patients with a MAGNIMS score of 0 (HR 1.96, *P* = 0.0044). Furthermore, an analysis performed on a cohort form the MSBase registry found that the prognostic value of the MAGNIMS score did not differ among patients on IFNβs, GA, fingolimod, and natalizumab (42). MAGNIMS score of 2 was associated with a significant increase in the risk of disability worsening when compared with MAGNIMS score of 0 (HR 1.72, *P* = 0.001).

The results of these studies are reflected in the joint EAN and ECTRIMS guidelines for pharmacological treatment of pwMS. It is recommended to offer a more efficacious drug for pwMS treated with DMT who experience relapses and/or disability progression and/or MRI activity at 6 to 12 months after treatment commencement (18). Therefore, after the initiation of the DMT, pwMS should be clinically and radiologically monitored for signs of breakthrough disease activity. As signs of the MS activity during early treatment with DMT carry greater risk of the future disability, a high-efficacy treatment should be offered.

Taking into consideration published studies, as well as EAN/ECTRIMS guidelines (18), our recommendations for switching from platform to high-efficacy therapies in people with relapsing MS are as follows:

### Recommendation for switching from platform to high-efficacy therapies (natalizumab/S1PR modulators/alemtuzumab/ocrelizumab/cladribine/ofatumumab) in people with relapsing multiple sclerosis:

pwMS on the treatment with platform therapies with EDSS≤7,0 and clinical and/or MRI activity defined as one of the following:

a. ≥1 relapse

b. ≥3 new T2 lesions

## Conclusions

In this review article, we presented the Croatian Neurological Society guidelines for the treatment of relapsing MS. The main goal of these guidelines is to enable the start of MS treatment as soon as diagnosis has been established, and to enable access to high-efficacy DMT as soon as needed.

Currently, in European clinical practice, treatment choice is often influenced by limited access to high-efficacy DMTs due to restrictions imposed by reimbursement bodies. In Europe, approximately 20% of pwMS gain access to the most innovative treatments, with lower proportions in the eastern European countries (3–4%) (8). The most obvious reason for this inequity are pharmacoeconomic and budget impact considerations that usually do not take into account long-term effectiveness and non-DMT costs associated with MS. In fact, long-term pharmaco-economic assessments may demonstrate societal benefits of early and unrestricted access to high-efficacy DMT. Further efforts should be made to facilitate access to high-efficacy DMT with a freedom of choice of an appropriate therapy for expert physicians and pwMS. Furthermore, the improvement of access to DMT achieved by implementation of national treatment guidelines in Croatia can serve as an example for national neurological societies from other Eastern European countries to persuade payers to enable early and unrestricted treatment of pwMS.
